# Endovascular treatment of traumatic pseudoaneurysm of the ileal branch of the superior mesenteric artery in a 9-year-old girl: Case report and literature review

**DOI:** 10.1097/MD.0000000000037978

**Published:** 2024-04-26

**Authors:** Hyung Jun Kwon, Jung Guen Cha, Jinyoung Park

**Affiliations:** a Department of Surgery, School of Medicine, Kyungpook National University, Kyungpook National University Hospital, Daegu, South Korea; b Department of Radiology, School of Medicine, Kyungpook National University, Kyungpook National University Hospital, Daegu, South Korea.

**Keywords:** case report, children, endovascular, superior mesenteric artery, visceral artery pseudoaneurysm

## Abstract

**Rationale:**

Visceral artery aneurysm is a rare and potentially fatal vascular condition that typically affects the superior mesenteric or inferior mesenteric arteries, the splenic, hepatic, and celiac arteries, as well as their branches. Visceral artery aneurysms can usually be treated using endovascular intervention, open surgery, or percutaneous thrombin injection.

**Patient concerns:**

A 9-year-old girl was admitted to our trauma center with abdominal and bilateral leg pain after a car accident involving a head-on collision.

**Diagnosis:**

Abdominal computed tomography (CT) showed bowel herniation through a muscle defect in the left lateral abdominal wall. There was a small amount of fluid around the liver and spleen, mild thickening of the small bowel wall, and infiltration in the small bowel mesentery, indicating the possibility of small bowel injury.

**Interventions:**

Emergent exploratory laparotomy was performed. After resection of the ischemic parts of the terminal ileum and sigmoid colon, intestinal continuity was reestablished. Primary repair was performed on a traumatic left lateral abdominal wall hernia. She recovered well postoperatively without any complications. A follow-up abdominal CT scan after 2 months showed a pseudoaneurysm of the ileal branch of the superior mesenteric artery. Despite the absence of any gastrointestinal symptoms, the pseudoaneurysm was treated by endovascular intervention using numerous coils because of the significant risk of delayed rupture or massive bleeding.

**Outcomes:**

Follow-up abdominal CT scan after 6 months showed complete occlusion and resorption of the pseudoaneurysm.

**Lessons:**

Although it is technically challenging, endovascular coil embolization may be a feasible technique in children with traumatic visceral artery pseudoaneurysms without complications.

## 1. Introduction

Visceral artery aneurysm is a relatively uncommon and potentially fatal vascular disease that typically affects the splenic, hepatic, celiac, superior mesenteric, or inferior mesenteric arteries and their branches with a documented prevalence of 0.1% to 2%.^[1–4]^ Visceral artery aneurysm is a very rare disease in the pediatric population and only a few cases have been reported to date.^[5–8]^ With the increased use of modern imaging modalities, including computed tomography (CT) angiography and magnetic resonance angiography, visceral artery aneurysms are increasingly being detected in asymptomatic patients, allowing early treatment and better outcomes.^[9]^ The treatment options for visceral artery aneurysms include percutaneous thrombin injection, open surgical approach, or endovascular approach. Due to recent technological advances, endovascular interventions are increasingly being used for the treatment of visceral artery aneurysms. Transcatheter embolization with coils or glue and covered stent placement are the commonly used endovascular procedures.^[2]^ The choice of procedure depends on the anatomic location of the affected artery and the size and type of the aneurysm. We report a traumatic pseudoaneurysm of the ileal branch of the superior mesenteric artery (SMA) in a 9-year-old girl, which was successfully treated by endovascular coil embolization.

## 2. Case report

**Figure 1.**
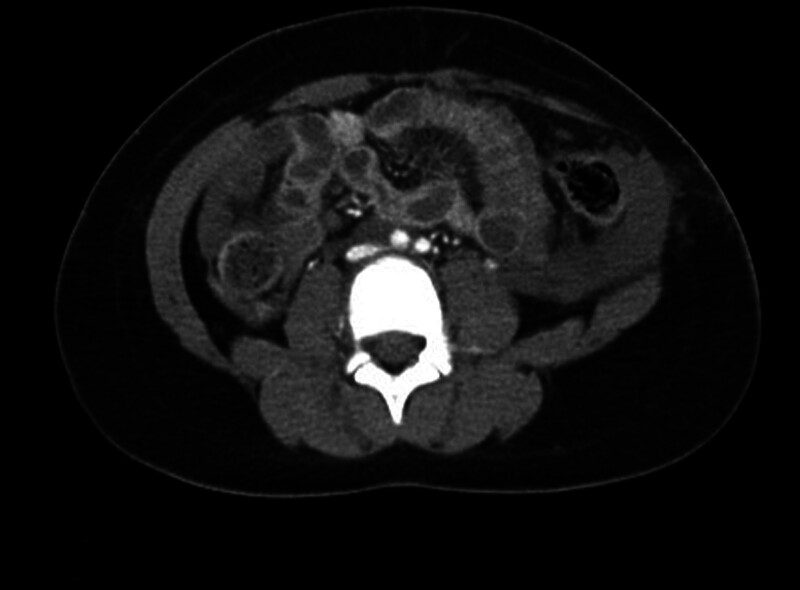
Initial abdominal computed tomography (CT) showing bowel herniation through the muscular defect in the left lateral abdominal wall. A small amount of fluid around the liver and spleen, mild thickening of the small bowel wall, and infiltration in the small bowel mesentery are also observed, indicating the possibility of small bowel injury.

**Figure 2.**
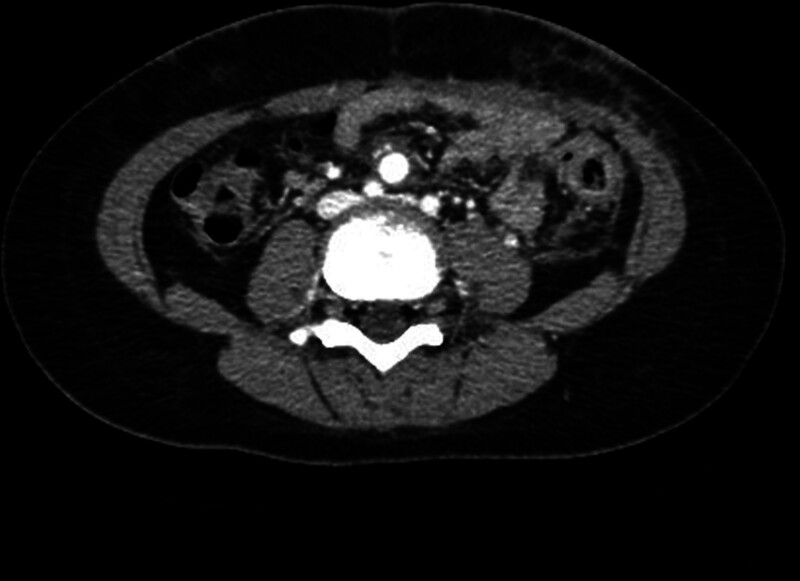
Follow-up abdominal CT scan after 2 months showing a highly enhancing round lesion (1 cm) in the mid-abdomen, suggesting a pseudoaneurysm of the ileal branch of the superior mesenteric artery.

**Figure 3.**
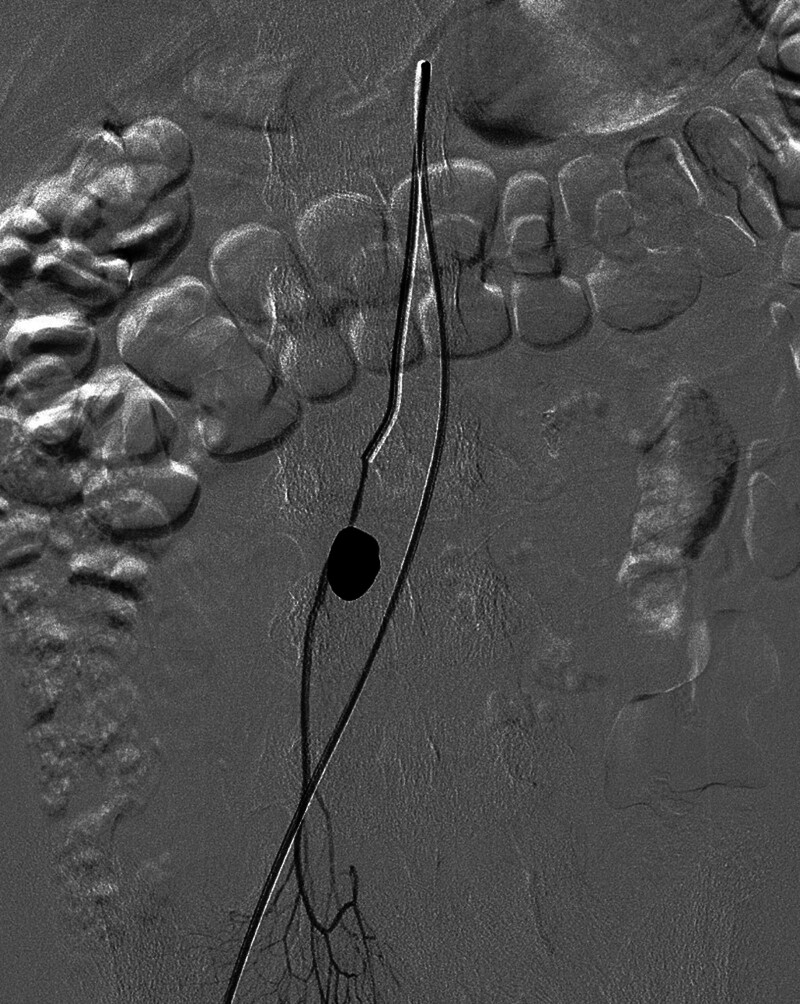
Digital subtraction angiography with selective catheterization of the superior mesenteric artery (SMA) performed via the right common femoral artery shows focal rupture and pseudoaneurysm at the mid portion of the ileal branch of the SMA.

**Figure 4.**
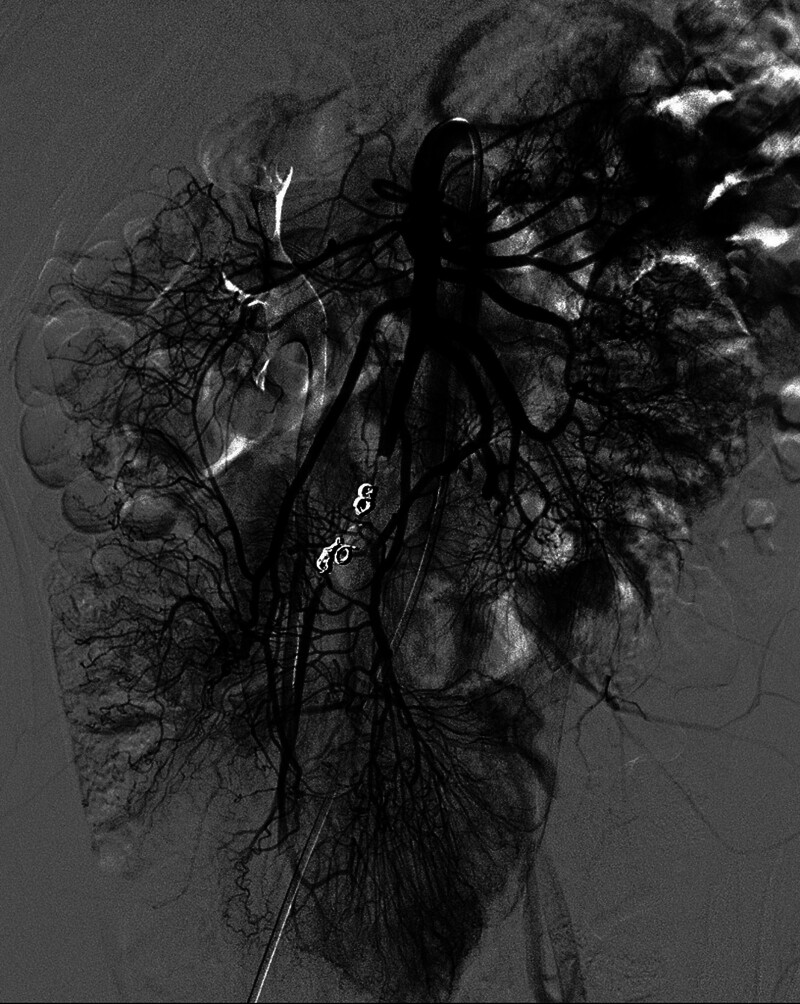
The ileal branch pseudoaneurysm was excluded by occluding the culprit vessel before and after the rupture point using detachable coils, while the distal blood flow was sustained by the surrounding collaterals.

A 9-year-old girl was admitted to our trauma center with pain in the abdomen and both legs after being injured in a car accident involving a head-on collision. She was wearing a seat belt in the passenger seat at the time of the accident. Her past medical history was unremarkable. She complained of moderate pain in the whole abdomen, both legs, and face. Her vital signs at the time of arrival were as follows: blood pressure, 93/61 mm Hg; heart rate, 139 beats per minute; respiratory rate, 20 breaths per minute; body temperature, 36.1 °C. Physical examination revealed mild tenderness in the whole abdomen. Initial laboratory investigations showed normal platelet count, erythrocyte sedimentation rate, and C-reactive protein level. However, her white blood cell count was elevated (24,410/mm^3^) and her hemoglobin level was low (9.4 g/dL). Her serum enzyme levels were as follows: aspartate transaminase, 100 U/L; alanine transaminase, 39 U/L; serum amylase, 373 U/L; creatine phosphokinase, 3660 U/L; lactate dehydrogenase, 709 U/L; myoglobin, 3164 ng/mL. Abdominal CT showed bowel herniation through a muscular defect in the left lateral abdominal wall. In addition, there was a small amount of fluid around the liver and spleen, mild thickening of the small bowel wall, and infiltration in the small bowel mesentery, indicating the possibility of small bowel injury (Fig. 1). An emergent exploratory laparotomy was performed. Intraoperative examination revealed a 30 cm long ischemic segment in the terminal ileum with rupture of the adjacent mesentery. This ischemic segment was resected and the intestinal continuity was restored using the Gambee suture technique. A 5 cm long deserosalizing type injury of the sigmoid colon with mesocolonic rupture was also resected and the intestinal continuity was restored using the Gambee suture technique. The traumatic left lateral abdominal wall hernia was primarily repaired layer by layer using a 2-0 absorbable interrupted suture. She recovered well postoperatively without any complications. Follow-up abdominal CT performed after 2 months showed a highly enhancing round lesion (size: 1 cm) in the mid-abdomen, which was suspected to be a pseudoaneurysm of the ileal branch of the SMA (Fig. 2). Despite the lack of any gastrointestinal symptoms, a multidisciplinary team, including a vascular surgeon and interventional radiologist, decided to perform an endovascular treatment due to the high risk of delayed rupture or massive bleeding after consulting the parent. Digital subtraction angiography with selective catheterization of the SMA was performed via the right common femoral artery. It revealed focal rupture and pseudoaneurysm at the mid portion of the ileal branch of the SMA (Fig. 3). The ileal branch pseudoaneurysm was excluded by occluding the culprit vessel before and after the rupture point using detachable coils, while distal blood flow was sustained by the surrounding collaterals (Fig. 4). The postprocedural course was uneventful. Follow-up abdominal CT scan after 6 months showed complete occlusion and resorption of the pseudoaneurysm with no relevant ileal loop ischemia.

## 3. Discussion

Visceral artery aneurysm is a relatively uncommon and potentially fatal vascular disease that commonly affects the splenic, hepatic, celiac, superior mesenteric, or inferior mesenteric arteries and their branches with a documented prevalence of 0.1% to 2%.^[1–4]^ There have only been a few documented reports of visceral artery aneurysms in children.^[5–8]^ True and pseudoaneurysms are both classified as visceral artery aneurysms. True visceral artery aneurysms affect all 3 layers of the vessel wall, i.e., tunica intima, media, and adventitia. Visceral artery pseudoaneurysms or false aneurysms occur due to damage to the intimal and medial layer, leading to the collection of blood on the outside of the artery, but which is lined or encased by the intact adventitia or perivascular surrounding tissue.

True visceral artery aneurysms are associated with connective tissue diseases (such as fibromuscular dysplasia, Ehlers-Danlos syndrome, and Marfan syndrome) as well as degenerative or atherosclerotic changes. On the other hand, the most common causes of visceral artery pseudoaneurysms are infections, iatrogenic injuries, trauma, and local inflammation.

Patients with visceral artery aneurysm may present with acute abdominal pain, nausea, vomiting, postprandial intestinal angina, gastrointestinal bleeding, and retroperitoneal or mesenteric hemorrhage. However, many cases are asymptomatic until the rupture or thrombosis of the aneurysm, resulting in significant bleeding and perhaps fatal consequences.

Visceral artery aneurysms are being more often diagnosed in asymptomatic patients due to the increasing use of contemporary imaging modalities including magnetic resonance angiography and CT angiography. This enables earlier treatment and improved outcomes.^[9]^ Color Doppler ultrasonography, CT, magnetic resonance imaging, and digital subtraction angiography can all be used to diagnose visceral artery aneurysms. Since ultrasonography is easy to use, quick, and radiation-free, it can be performed on children presenting with abdominal pain. CT vascular imaging and 3-dimensional post-processing can delineate the vascular course, the caliber of the visceral artery and its branches, and the communication between the aneurysm and the blood vessels. Arteriography can also help locate the aneurysm and facilitate preoperative treatment plan. Concomitant use of color Doppler ultrasonography and contrast-enhanced CT can confirm the diagnosis of aneurysm of the SMA and its collateral branches, enabling prompt management.

Much of the contemporary literature on mesenteric branch aneurysms is limited to small case series and does not distinguish between true aneurysms and pseudoaneurysms. Visceral artery pseudoaneurysms are at a higher risk of rupture than true aneurysms. According to guidelines published by the Society of Vascular Surgery, adults with jejunal and ileal artery aneurysms that have a maximal diameter of more than 2 cm should undergo elective intervention; those with symptomatic or ruptured aneurysms of any size should undergo emergent intervention; and all mesenteric branch vessel pseudoaneurysms, regardless of size or symptoms, should undergo emergent intervention.^[10]^

Visceral artery aneurysms are usually treated with either endovascular or open surgical procedures. Open surgical procedures include simple ligation or resection of aneurysm and end-to-end anastomosis, aneurysmorrhaphy in case of saccular aneurysm, bypass surgery with a venous interposition graft, and intestinal resection. Due to recent advances, endovascular treatment is increasingly being used to treat even anatomically complex visceral artery aneurysms. Endovascular treatment includes transcatheter embolization with coils, Onyx, and glue or the insertion of covered stents. Endovascular treatment can verify the location of the aneurysm and collateral blood flow. It is associated with a relatively short hospital stay, is easily accessible, and poses a lower risk for patients who are not suitable candidates for surgery. Although not all visceral artery aneurysms can be treated via the endovascular approach, the choice of treatments depends on several factors, including the size and location of the aneurysm, the patient’s general condition, and the experience and expertise of the interventional radiologist. Batagini et al reported similar rates of technical and clinical success with open and endovascular treatment for visceral artery aneurysms.^[3]^ Preserving the major tributaries is a crucial consideration when considering endovascular treatment for SMA aneurysms extending beyond the proximal few centimeters. Loss of these mesenteric peripheral collateral blood supplies due to endovascular treatment may lead to serious complications, such as intestinal necrosis, perforation, and late stricture, necessitating reoperation; therefore, surgical treatment should be considered in such cases. A few factors should be considered while diagnosing and treating visceral artery aneurysms in children, even though there are no appreciable differences between adult and pediatric patients. Firstly, in contrast to adults, endovascular procedures in children require general anesthesia or sedation, and associated complications may arise. Second, the smaller vessels in pediatric patients may pose a technical challenge that needs to be addressed by an experienced radiologist.

## 4. Conclusion

Endovascular coil embolization may be a feasible technique in children with traumatic visceral artery pseudoaneurysms without complications.

## Author contributions

**Conceptualization:** Hyung Jun Kwon, Jung Guen Cha, Jinyoung Park.

**Formal analysis:** Hyung Jun Kwon, Jinyoung Park.

**Writing – original draft:** Hyung Jun Kwon, Jinyoung Park.

**Writing – review & editing:** Hyung Jun Kwon, Jung Guen Cha, Jinyoung Park.
